# Helicobacter Pylori Eradication Therapy: Still a Challenge

**DOI:** 10.7759/cureus.14872

**Published:** 2021-05-06

**Authors:** Muhammad Hafeez, Zafar A Qureshi, Abdul L Khattak, Farrukh Saeed, Asma Asghar, Khalid Azam, Muhammad A Khan

**Affiliations:** 1 Gastroenterology, Pak Emirates Military Hospital, Rawalpindi, PAK; 2 Medicine, Combined Military Hospital, Lahore, PAK; 3 Gastroenterology, Combined Military Hospital, Lahore, PAK; 4 Pulmonology, Combined Military Hospital, Lahore, PAK; 5 Physiology, Army Medical College, Rawalpindi, PAK

**Keywords:** drug regimens for h. pylori, helicobacter pylori, h pylori treatment challenges

## Abstract

Introduction

Helicobacter pylori (H. pylori) infection is prevalent worldwide. H. pylori therapies' adverse effects can contribute to noncompliance among patients. This study aimed to assess the association between compliance to H. pylori eradication therapy and adverse effects using various drug regimens.

Method

We conducted an observational study from September 2017 to February 2020 in two tertiary care hospitals in patients with dyspeptic symptoms. H. Pylori detection was done by histopathological examination of gastric mucosa during upper gastrointestinal endoscopy or stool for H. pylori antigen. Patients with positive results were randomly assigned one of the nine different regimens consisting of a combination of proton pump inhibitors along with at least two antibiotics. The antibiotics used in different combinations were amoxicillin, clarithromycin, metronidazole, doxycycline, levofloxacin, and bismuth sulfate. The treatment groups received standard triple therapy with and without probiotics, sequential, concomitant, levofloxacin-based triple therapy, or sequential and bismuth-based quadruple treatments. All treatments were given for two weeks. At the end of the treatment period, patients were interviewed about completing treatment and any adverse effects they may have experienced during therapy. Data were analyzed using IBM Statistical Package for the Social Sciences (SPSS) Statistics for Windows, Version 22.0 (Armonk, NY: IBM Corp.).

Results

A total of 250 patients were included in the study (62% males, 38% females) with a mean age of 37 years ± 13 years (range 12-84 years). Most patients completed the treatment regimen (80.4%), and 19.6% did not complete treatment because of adverse effects (p<0.005). The levofloxacin-based, concomitant, and standard triple regimen with probiotic treatments had the highest tolerance (≥85%). Common adverse effects were abdominal and epigastric pain (11%), alteration of taste, and diarrhea (6.5%).

Conclusion

H. pylori eradication therapy is always a challenge. Patient compliance to the treatment can only be ensured by medicines with fewer adverse effects. In our study, levofloxacin-based triple, concomitant, and standard triple regimens with probiotics are maximally acceptable treatments.

## Introduction

More than half of the world's population is suffering from infection with Helicobacter pylori (H. pylori), and given the improvement of sanitary standards, the prevalence of H. pylori infection varies widely in different parts of the world [1-3]. H. pylori is a Gram-negative bacterium that colonizes gastric mucosa. Its detection in 1983 revolutionized peptic ulcer treatment [4]. H. pylori has oral-to-oral and fecal-oral transmission. Parents and siblings play an important role in transmission [5].

The clinical features of H. pylori infection vary from asymptomatic to mucosa-associated lymphoid tissue lymphoma. Common conditions associated with H. pylori are acid peptic disease, idiopathic thrombocytopenic purpura, iron deficiency, and vitamin B12 deficiency [6]. H. pylori eradication therapy is recommended in patients who test positive [7]. Because H. pylori infections are challenging to treat, combination therapy of at least two antibiotics and proton pump inhibitors (PPIs) are recommended [8].

The most common adverse effects of antibiotics are related to the GI system like abdominal pain, epigastric pain, diarrhea, constipation, and metallic taste [9]. Other adverse effects are headaches with or without vomiting. Intolerance to the drugs due to their adverse effects leads to noncompliance with the treatment, culminating in treatment failure and resistance to these drugs. Several studies have been conducted on effective medicines, treatment outcomes, and possible adverse effects, but very few have quantified patients' ability to complete the treatment based on adverse effects [10-12]. We conducted this study to determine the association between patient compliance to H. pylori eradication therapy and adverse effects using various drug regimens.

## Materials and methods

We conducted this observational study in Combined Military Hospital (CMH) Multan and Pak Emirates Military Hospital (PEMH) Rawalpindi tertiary care hospitals from September 2017 to February 2020. The study design was approved by the ethical committee of the hospitals. We used consecutive convenient sampling. All patients with dyspepsia due to H. pylori infections were included in the study after providing written informed consent. We excluded patients with dyspepsia secondary to other causes, patients treated with ongoing PPIs, and patients treated with antibiotics with anti-H. pylori effects. Pregnant ladies were also excluded due to the possible teratogenic effects of drugs. H. pylori was detected via histopathological examination of the gastric mucosal biopsy specimen taken during an upper GI endoscopy or via stool examination. Gastric biopsy specimens were stained with hematoxylin and eosin with and without Giemsa staining to detect spiral-shaped H. pylori organisms on histopathology. The detection of H. pylori antigens in stool used an enzyme immunoassay qualitative or semiquantitative multiple-step method. Patients were assigned into nine groups randomly that were computed based on as specified in Table 1, and each group underwent a different treatment to observe for adverse effects and compliance.

**Table 1:** Drug regimens used in the study PPI: proton pump inhibitor

Serial no.	Drug regimens
1	PPI + clarithromycin + amoxicillin (standard triple treatment)
2	PPI + amoxicillin + metronidazole (standard triple treatment)
3	PPI + bismuth + doxycycline + amoxicillin + nitroimidazole (bismuth-based quadruple treatment)
4	PPI + clarithromycin + amoxicillin + nitroimidazole (concomitant treatment)
5	PPI + clarithromycin + amoxicillin + nitroimidazole (concomitant treatment)
6	PPI + amoxicillin 7 days followed by PPI + clarithromycin + nitroimidazole 7 days (sequential treatment)
7	Amoxicillin 7 days followed by PPI + amoxicillin + nitroimidazole 7 days (sequential treatment)
8	PPI + levofloxacin + amoxicillin 14 days (levofloxacin-based triple treatment)
9	PPI + clarithromycin + amoxicillin + probiotics (probiotic supplemented triple treatment)

After two weeks of treatment, participants were interviewed on whether they completed the treatment and, if not, why they did not complete the treatment. 

The data were analyzed using IBM Statistical Package for the Social Sciences (SPSS) Statistics for Windows, Version 22.0 (Armonk, NY: IBM Corp.). Descriptive data were calculated as mean, standard deviation, and percentage. We used the Chi-square test to analyze quantitative data, and p≤0.05 was considered statistically significant.

## Results

A total of 250 participants were included in the study, with 154 male patients (61.6%) and 96 female patients (38.4%). The study population's mean age was 36.7 years ± 13.2 years. Table 2 shows the correlation between adverse effects and treatment compliance. Of 250 patients, 135 (54.0 %) had adverse effects; 87 (64.4%) patients had mild-to-moderate adverse effects (p=0.000) and in 49 patients (19.6%), the adverse effects were so severe that they were unable to complete the treatment (p<0.001; odds ratio, 62.9; 95% confidence interval 8.5-464.7). Compliance was better in males, i.e., 130 (84.40%) as compared to females 71 (74%) which was significant statistically (p=0.04). The association between treatment compliance and adverse effects is also statistically significant, and the odds of compliance are 62.9 times higher in patients who did not experience adverse effects compared to those who experienced adverse effects.

**Table 2:** Association of adverse effects with treatment compliance PPI: proton pump inhibitor

Adverse effect	Treatment compliance		Total	p-Value	Odds ratio with 95% confidence interval
	Yes	No			
No	114 (99.1%)	1 (0.9%)	115 (46.0%)	<0.001	62.9 (8.5-464.7)
Yes	87 (64.4%)	48 (35.6%)	135 (54.0%)		
Total	201 (80.4%)	49 (19.6%)	250		

A descriptive analysis of different types of adverse effects and cross-tabulation of each with treatment compliance is shown in Table 3. The lowest compliance occurred in patients with diarrhea, followed by patients reporting abdominal/epigastric pain. Diarrhea was found in 26 cases (10.4%), of whom half (50%) could not complete the H. pylori eradication therapy. Compliance was relatively much better with patients experiencing other adverse effects. Nausea with and without vomiting was observed in 1.6% of participants. Other adverse effects noted with <1% frequency were dizziness, constipation, burning feet, anxiety, restlessness, and irritability in different drug combinations. The frequency comparison of patient compliance in various categories of adverse effects is statistically significant (p<0.001).

**Table 3:** Association of various adverse effects with treatment compliance

Detail of adverse effects	Treatment compliance		Total	p-Value
	Yes	No		
Abdominal/epigastric pain	22 (52.4%)	20 (47.6%)	42 (16.8%)	<0.001
Diarrhea	13 (50.0%)	13 (50.0%)	26 (10.4%)	
Alteration in taste	32 (72.7%)	12 (27.3%)	44 (17.6%)	
Headache with or without vomiting	10 (100.0%)	0 (0.0%)	10 (4%)	
Miscellaneous	10 (76.9%)	3 (23.1%)	13 (5.2%)	

Table 4 presents the frequency and percentage of patients with and without adverse effects in different drug regimens. Drug regimen was significantly associated with adverse effects (p=0.002). Table 5 shows a detailed descriptive analysis of compliance and side effects in various drug regimens. Patient compliance in different drug regimen groups was not statistically significant (p=0.62). A comparison of treatment compliance in different genders showed poorer compliance in female participants than male participants (26% vs 15.60%; p=0.05). The frequency comparison of patients with compliance among different drug regimen groups was statistically nonsignificant at a p-value of 0.62.

**Table 4:** Association of drug regimen with adverse effects PPI: proton pump inhibitor

Drug regimen	Side effects		p-Value
	Yes	No	
PPI + clarithromycin + amoxicillin	30 (71.4%)	12 (28.6%)	0.002
PPI + amoxicillin + metronidazole	11 (55.0%)	9 (45.0%)	
PPI + bismuth + doxycycline + amoxicillin + nitroimidazole	10 (43.5%)	13 (56.5%)	
PPI + clarithromycin + amoxicillin + nitroimidazole	23 (54.8%)	19 (45.2%)	
PPI + amoxicillin 7 days followed by PPI + clarithromycin + nitroimidazole 7 days	13 (65.0%)	7 (35.0%)	
PPI + amoxicillin 7 days followed by PPI + amoxicillin + nitroimidazole 7 days	12 (60.0%)	8 (40.0%)	
PPI + levofloxacin + amoxicillin 14 days	11 (37.9%)	18 (62.1%)	
Levofloxacin + PPI + amoxicillin 7 days followed by fluoroquinolones+ PPI + nitroimidazole	22 (64.7%)	12 (35.3%)	
PPI + clarithromycin + amoxicillin + probiotics	3 (15.0%)	17 (85.0%)	

**Table 5:** Descriptive analyses of compliance and side effects in various drug regimens PPI: proton pump inhibitor

Drug regimen	Compliance	Side effects	Frequency	Percent (%)
PPI + clarithromycin + amoxicillin; Total: 42	Yes; Total: 32 (76.2%)	No side effect	11	34.4
		Abdominal/epigastric pain	5	15.6
		Diarrhea	1	3.1
		Alteration in taste	12	37.5
		Miscellaneous	3	9.4
	No; Total: 10 (23.8%)	No side effect	1	10.0
		Abdominal/epigastric pain	6	60.0
		Diarrhea	1	10.0
		Alteration in taste	2	20.0
PPI + amoxicillin + metronidazole; Total: 20	Yes; Total: 16 (80.0%)	No side effect	9	56.3
		Abdominal/epigastric pain	2	12.5
		Diarrhea	1	6.3
		Alteration in taste	2	12.5
		Headache with or without vomiting	2	12.5
	No; Total: 4 (20.0%)	Abdominal/epigastric pain	1	25.0
		Diarrhea	2	50.0
		Alteration in taste	1	25.0
PPI + bismuth + doxycycline + amoxicillin + nitroimidazole; Total: 23	Yes; Total: 17 (73.9%)	No side effect	13	76.5
		Abdominal/epigastric pain	1	5.9
		Diarrhea	1	5.9
		Headache with or without vomiting	2	11.8
	No; Total: 6 (26.1%)	Abdominal/epigastric pain	3	50.0
		Diarrhea	2	33.3
		Miscellaneous	1	16.7
PPI + clarithromycin + amoxicillin + nitroimidazole; Total: 42	Yes; Total: 36 (85.7%)	No side effect	19	52.8
		Abdominal/epigastric pain	2	5.6
		Diarrhea	3	8.3
		Alteration in taste	6	16.7
		Headache with or without vomiting	3	8.3
		Miscellaneous	3	8.3
	No; Total: 6 (14.3%)	Diarrhea	3	50.0
		Alteration in taste	3	50.0
PPI + amoxicillin 7 days followed by PPI + clarithromycin + nitroimidazole 7 days; Total: 20	Yes; Total: 13 (65.0%)	No side effect	7	53.8
		Diarrhea	1	7.7
		Alteration in taste	4	30.8
		Headache with or without vomiting	1	7.7
	No; Total: 7 (35.0%)	Abdominal/epigastric pain	3	42.9
		Diarrhea	1	14.3
		Alteration in taste	3	42.9
PPI + amoxicillin 7 days followed by PPI + amoxicillin + nitroimidazole 7 days; Total: 20	Yes; Total: 16 (80.0%)	No side effect	8	50.0
		Abdominal/epigastric pain	5	31.3
		Diarrhea	2	12.5
		Alteration in taste	1	6.3
	No; Total: 4 (20.0%)	Abdominal/epigastric pain	3	75.0
		Alteration in taste	1	25.0
PPI + levofloxacin + amoxicillin 14 days; Total: 29	Yes; Total: 25 (86.2%)	No side effect	18	72.0
		Abdominal/epigastric pain	3	12.0
		Diarrhea	1	4.0
		Headache with or without vomiting	1	4.0
		Miscellaneous	2	8.0
	No; Total: 4 (13.8%)	Diarrhea	2	50.0
		Alteration in taste	1	25.0
		Miscellaneous	1	25.0
Levofloxacin + PPI + amoxicillin 7 days followed by levofloxacin + PPI + nitroimidazole; Total: 34	Yes; Total: 29 (85.3%)	No side effect	12	41.4
		Abdominal/epigastric pain	4	13.8
		Diarrhea	3	10.3
		Alteration in taste	7	24.1
		Headache with or without vomiting	1	3.4
		Miscellaneous	2	6.9
	No; Total: 5 (14.7%)	Abdominal/epigastric pain	1	20.0
		Diarrhea	2	40.0
		Alteration in taste	1	20.0
		Miscellaneous	1	20.0
PPI + clarithromycin + amoxicillin + probiotics; Total: 20	Yes; Total: 17 (85.0%)	No side effect	17	100.0
	No; Total: 3 (15.0%)	Abdominal/epigastric pain	3	100.0

## Discussion

One of the important contributors to any therapy's success is adherence and compliance to the treatment regimen. The same is true for patients undergoing H. pylori eradication therapy. Noncompliance is responsible for the failure of therapy in antibiotic-sensitive patients and can lead to antibiotic resistance. This can have long-term implications, and therefore, clinicians need to have a clear understanding of patients' reasons for noncompliance. This can be multifactorial, but adverse effects associated with the drugs play an important role. These factors have been discussed in several studies. In all these studies, intolerance to drugs comprises an important factor in treatment failure [13-15].

In our study, 50% of patients had adverse effects with the drug combinations of amoxicillin, metronidazole, and clarithromycin, and 24 % of patients taking levofloxacin with those antibiotics. This rate of adverse effects is the same as that reported in a meta-analysis by Fischbach et al., who reported that 50% of patients had adverse effects with one of the triple regimens [16].

The frequency of adverse effects in patients treated with clarithromycin was higher (74%) than those treated with metronidazole, amoxicillin, and PPI (55%), which was also higher than adverse effects in patients treated with bismuth-based quadruple therapy (43%). Luther et al. did not find any difference in these two regimens for compliance, tolerance, and efficacy [17]. GI adverse effects like nausea, vomiting, diarrhea, and abdominal pain were reported in 1% to 14% in the clarithromycin and azithromycin individual treatment groups, but the frequency was be increased (76%) when used in combination with other antibiotics. Medicines like macrolides and metronidazole can lead to a metallic taste in the mouth, and they are excreted in saliva through diffusion or carrier-mediated transport. This effect's proposed mechanism is drug-receptor interaction and a disturbance in the action potential propagation in afferent and efferent neurons [18]. We found an alteration of taste in 30.8% of patients receiving sequential therapy.

The levofloxacin-based triple and sequential treatments have an equal ratio of tolerance, adverse effects, and maximum completion rate. The unpleasant metallic taste (4%) and diarrhea (15%) experienced by patients receiving levofloxacin-based treatment were due to metronidazole and amoxicillin, respectively [19-21].

Probiotics are live microorganisms that are host-friendly and offer health benefits. Commonly used probiotic bacteria Lactobacillus and Bifidobacterium improve H. pylori eradication and reduce adverse effects [22-24]. With the addition of probiotics, the tolerance to the clarithromycin-based triple regimen increased to >85% in our study, and GI symptoms reduced from 76% in the standard triple regimen to 15% (p=0.002). Antibiotic-associated diarrhea has been documented with certain antibiotics like clindamycin, cephalosporin, and amoxicillin-clavulanate and occurs due to clostridium difficile bacterial overgrowth leading to pseudomembranous colitis [25].

Poor tolerance to H. pylori therapies was more common among female patients (26%) than male participants in our study (15.6%; p=0.05). Yokota et al. reported a marginally significant association between male gender and self-interruption of therapy, contrary to our results [26].

Our study had several important limitations and is not absolutely blind, and the design allowed patients to opt-out of a particular regimen based on past experience with any specific drug. In addition, we lack the availability of drug sensitivity testing. However, despite these limitations, our study is unique in that it compared noncompliance due to adverse effects among nine eradication regimens. Previously published studies were limited in comparing only three to five eradication regimens [27,28].

## Conclusions

H. pylori infection has long-term implications. To avoid antibiotic resistance in the future and improve patient compliance, clinicians need to clearly understand the factors associated with intolerance to the various regimens.

Levofloxacin-based triple, sequential, concomitant, and standard triple treatment with probiotics have maximum tolerance and compliance with a completion rate of more than 85% with lesser side effects. Sequential treatment of standard three antibiotics has the least completion rate of 65% because of severe metallic distaste. However, further studies are required to strengthen the study.
